# Distributed organization of a brain microcircuit analyzed by three-dimensional modeling: the olfactory bulb

**DOI:** 10.3389/fncom.2014.00050

**Published:** 2014-04-29

**Authors:** Michele Migliore, Francesco Cavarretta, Michael L. Hines, Gordon M. Shepherd

**Affiliations:** ^1^Department of Neurobiology, School of Medicine, Yale UniversityNew Haven, CT, USA; ^2^Institute of Biophysics, National Research CouncilPalermo, Italy

**Keywords:** olfactory glomeruli, mitral cells, granule cells, lateral inhibition, computational modeling

## Abstract

The functional consequences of the laminar organization observed in cortical systems cannot be easily studied using standard experimental techniques, abstract theoretical representations, or dimensionally reduced models built from scratch. To solve this problem we have developed a full implementation of an olfactory bulb microcircuit using realistic three-dimensional (3D) inputs, cell morphologies, and network connectivity. The results provide new insights into the relations between the functional properties of individual cells and the networks in which they are embedded. To our knowledge, this is the first model of the mitral-granule cell network to include a realistic representation of the experimentally-recorded complex spatial patterns elicited in the glomerular layer (GL) by natural odor stimulation. Although the olfactory bulb, due to its organization, has unique advantages with respect to other brain systems, the method is completely general, and can be integrated with more general approaches to other systems. The model makes experimentally testable predictions on distributed processing and on the differential backpropagation of somatic action potentials in each lateral dendrite following odor learning, providing a powerful 3D framework for investigating the functions of brain microcircuits.

## Introduction

Analyzing a brain system in its natural three-dimensional (3D) layout is an essential goal for discovering constraints critical for guiding reduction of its complexity and abstraction into more general theoretical representations. In choosing a brain system to reveal the types of higher functions that can emerge from these types of implementation, a few specific properties are highly desirable. There should be a relatively small set of cell types and total number. The cells should have a relatively simple network organization. They should be widely studied experimentally. It should be relatively easy to carry out and compare experiments across different species, including humans. The microcircuit should be involved in higher brain functions, such as encoding and recognition processes.

The olfactory bulb seems to be exquisitely tailored to fulfill these properties: in one synapse, an external signal goes from sensory neurons to olfactory cortex. The entire system is composed of only some 100,000 principal cells and a few millions of interneurons. It is one of the most studied systems (Shepherd et al., 2004); similar experiments can be carried out and compared across species (Murthy, 2011); and it is an essential stage for odor recognition processes (Mori and Takagi, 1975; Cleland and Linster, 2005; Wachowiak and Shipley, 2006). The output of the principal neurons, mitral/tufted cells, is controlled mainly through synapses between the dendrites of the mitral cells and their main interneuron, the granule cells (GC) (Yokoi et al., 1995). These dendrodendritic synapses have the advantage that they constitute one of the clearest examples of synaptic organization for local information processing in the nervous system (Rall et al., 1966; Rall and Shepherd, 1968).

How the mitral-granule cell microcircuit carries out sensory processing in the olfactory bulb has been investigated experimentally in terms of odor discrimination and dynamics of cell responses, usually in single cells or in small randomly selected sets of cells (e.g., Shusterman et al., 2011; Smear et al., 2011). However, the functional effects of network self-organization remain difficult to understand and to explore experimentally.

To aid in solving this problem, we have previously constructed a large-scale one-dimensional model of the olfactory bulb (Yu et al., 2013) to analyse how the spatio-temporal dynamics of lateral inhibition produce glomerular-related cell clusters during presentation of a series of relatively simple monomolecular odors. Here, we extend that approach with a novel 3D model for processing natural odorants. This is particularly important, since complex natural odors activate a large portion of the olfactory bulb with dense representation (Vincis et al., 2012). We postulate that under these conditions even a large network organization with canonical simplified neurons or abstract representations (Davison et al., 2003; Luo et al., 2010) or our previous models limited to 1D (Migliore et al., 2007, 2010; Migliore and Shepherd, 2008; McTavish et al., 2012; Yu et al., 2013), cannot solve the problem of understanding how odor discrimination is carried out efficiently by the actual neurons and their microcircuit connections. Using realistic 3D inputs, cell morphologies, and network connectivity, the model makes experimentally testable predictions on distributed processing of the odor input and on differential backpropagation of somatic action potentials in each lateral dendrite following odor learning. It introduces a powerful 3D approach of general applicability by computational modelers for investigating the functions of brain microcircuits.

## Methods

For the purpose of this work, we considered the activity of 127 glomeruli in a ≈2 mm^2^ dorsal area of the olfactory bulb (about 10% of the entire system). To constrain the model inputs with experimental data, we used the activity level from intrinsic optical imaging obtained for each of the 127 glomeruli during presentation of natural odors (from Vincis et al., 2012). The raw data from 20 odors (provided by A. Carleton) were normalized and directly used to set the peak conductance of synaptic inputs on the mitral cell tufts, assuming preprocessing by local glomerular circuits (reviewed in Linster and Cleland, 2009) To generate synthetic mitral cells (see Results), we analyzed 8 3D reconstructions of mitral cells, recently obtained by Igarashi et al. (2012) with full visualization of the entire dendritic trees. An initial implementation of the model network is described in Migliore et al. (2013).

### Electrophysiological and morphological properties

The network consists of 635 synthetic mitral cells (5 per glomerulus) connected to a variable number of GC (13,260–69,013, according to the connectivity rule, see later). All cells were composed of 20 μm membrane segments. Using a smaller (10 μm) segment size did not result in qualitatively different results (not shown). For mitral cells, uniform passive properties were used, with Ra = 150 Ωcm, and τ_m_ = 7 ms, in agreement with recent experimental findings (O'Connor et al., 2012) at physiological temperature. For each synthetic mitral cell, the shape of the soma was randomly chosen from the 8 reconstructions, whereas the lateral dendrites were generated as explained in Results (see later). The range and type of distribution chosen to set many parameters defining individual cell morphology and location are reported in Table 1, and described in more detail in the following paragraphs. A random number of (4–9) lateral dendrites arose from the soma with an initial random diameter, *D*_*i*_, of 3–5 μm, tapering with distance from the soma as *D*_*i*_ − 2.6 · exp(−0.0013 · *d*) to a minimum of 0.3 μm. For the apical and tuft dendrites, we used data from the reconstructions and the constraints of our laminar model of the olfactory bulb to set the range of values for diameter and length, which were also constrained to reproduce several experimental findings on the electrophysiological properties for mitral cells (Chen et al., 2002; Debarbieux et al., 2003). Briefly, non-bifurcating apical dendrites were modeled with 4–5 μm diameters and a length in the range of 420–520 μm (depending on the relative glomerulus location). To take into account the tuft morphology, a random number (between 4 and 6) of 0.8 μm diameter dendrites (40–60 μm long) were added at the tip of the apical dendrites. GC were modeled with a soma and a radial dendrite representing the dendritic tree. Its length was in the range 350–750 μm, according to the actual location of the soma in the Granule Cell Layer (GCL), and allowed the dendrite to span the entire EPL layer. All passive and active properties, already validated against experimental findings, were taken from our previous model (Yu et al., 2013, ModelDB acc.n.144570). Briefly, in mitral cells, Na, KA-, and KDR-type conductances were uniformly distributed over the entire dendritic tree. In GC, Na, and KA channels were distributed throughout whereas KDR was present only in the soma. Simulations were carried out at 35°C, with resting potential set at −65 mV for all cells.

**Table 1:** **Range and distribution of selected model parameters**.

	**Range**	**Type of distribution**
**MORPHOLOGICAL PARAMETERS**		
Number of MC apical dendrites	1	Constant
Diameter of MC apical dendrites	4–5 μm	Random, uniform; no tapering
Number of tuft dendrites	4–6	Random integer, uniform
Length of tuft dendrites	40–60 μm	Random, uniform
Diameter of tuft dendrites	0.8 μm	Constant; no tapering
MC Soma location (Longitude)	MCL	Random, uniform
MC Soma location (Latitude)	±50 μm (from relative glom. projection)	Random, uniform
Number of lateral dendrites	5 (avr); limited to 4–9	Random integer, poisson
Initial diam. of lateral dendrites	3–5 μm	Random, uniform; tapering (see text)
GC soma location	25 μm voxels (inside GCL)	Constant
**SYNAPSE PARAMETERS (TYPICAL VALUES)**		
1 Syn per tuft dend. (odor)	Global peak = 0.65 nS	Random, uniform, ±30%
Syn. inputs activ. freq. (sniffing)	2–10 Hz	Random, uniform; synchronous
Peak exc. conduct. (MC->GC)	1 nS	Constant
Peak inh. conduct. (GC->MC)	10 nS	Constant
Phantom synapses activ. freq.	10 Hz	Poisson, asynchronous

We would like to stress that we have chosen not to include, at this stage, a number of additional ion channels and mechanisms that have been found in these cells. The reason for this choice is that the focus in implementing this model is to understand the basic processes underlying lateral and feedback inhibition in a full 3D network. As we have already explained in detail in our previous papers (Migliore et al., 2007, 2010; Yu et al., 2013) these mechanisms critically depend only on action potential generation and propagation in mitral cells and GC. Of course any additional current and cell types can be easily included in future studies aiming at a more comprehensive implementation of the intrinsic cell electrophysiological properties, investigation of the functional role of other membrane currents, or the contributions of other cell types.

### Synaptic properties

Network connectivity is presented and discussed in Results. The range and type of distribution chosen to set peak conductance and activation properties of synaptic inputs modeling odor presentation and reciprocal synapses are summarized in Table 1. As in our previous models (Migliore et al., 2010; Yu et al., 2013), dendrodendritic coupling between granule cell synapses and mitral cell secondary dendrites was implemented by connecting a GC synapse, containing the same proportion of AMPA and NMDA channels, with the appropriate compartment of mitral cell secondary dendrites containing GABA channels. To model odor stimulation, independent synaptic inputs in each of the tuft branches of the involved mitral cells were activated with a double exponential conductance change (20 and 200 ms rise and decay time, respectively). To model an odor presentation, the synaptic inputs were activated at a random frequency in the range of 2–10 Hz, corresponding to the range of natural sniffing frequency during explorative behavior in rats (Kepecs et al., 2007). The peak synaptic conductance of each input was randomly chosen from a normal distribution consistent with the relative experimental activation (see Table 1). In test simulations, additional random “phantom” excitatory synapses were added to the granule cell dendrites, to take into account activity from all the mitral cells that were not explicitly modeled. The number of phantom synapses was proportionally higher in GC closer to the edge of the modeled area. They were randomly (Poisson) and asynchronously activated at an average frequency of 10 Hz, and followed the same plasticity rule of normal synapses. A network with phantom synapses included 3,414,771 excitatory synapses on the GC, complemented by 707,216 inhibitory granule cell synapses on the mitral cell lateral dendrites. For the typical simulation discussed in this paper, phantom synapses were not used.

### Odor learning

During a typical simulation for odor learning, synaptic weights started at zero and, in response to odor input, to undergo Long-Term Potentiation or Long-Term Depression. The plasticity rule used for all simulations has been previously used (Migliore et al., 2007, 2010; Yu et al., 2013), and already validated against a number of experimental findings on synaptic cluster formation and mitral cell spike time distribution following single sniffs (see Yu et al., 2013). During preliminary tests we found that, for our model, a 40 s odor presentation was sufficient to stabilize synaptic weights and cell activity, which was used for all simulations unless noted otherwise. It should be stressed that this time frame is only a convenient choice allowing the synaptic weights to reach a relatively stable configuration within a reasonable computation time. We are not currently interested in implementing and studying the actual temporal dynamics of odor learning as it occurs *in vivo* but, rather, the post-learning functional consequences of the olfactory bulb network self-organization on odor perception and recognition. From this point of view, we have previously noted, shown, and discussed in detail (Migliore et al., 2007, 2010) that the results using this rule are robust and do not depend on the specific choice for the functional form or time frame used to update the synaptic weights.

### Computational issues

All simulations were carried out with a fully integrated NEURON+Python parallel environment (NEURON v7.3, Hines and Carnevale, 1997) on a BlueGene/Q IBM supercomputer (CINECA, Bologna, Italy). The model and simulation files will be available for public download under the ModelDB section of the Senselab database suite (http://senselab.med.yale.edu, acc.n. 151681). Under typical control conditions, the model network was composed of a system of 31,152,052 differential equations corresponding to the different state variables of the system, i.e., voltages, gate variables, and synaptic states. In a typical 40 sec simulation, such as that discussed later, 745,104,507 spikes were generated in the 4,725,472 membrane segments, for a total run time of about 9 h using 2048 MPI processes. More detailed information on the simulation parameters and execution vs. communication times is reported in Table 2. A minimum partition size of 64 nodes with 32 processes per node seemed to result in minimum wait times in the job queue. Although run time was reduced by a factor of 2 when 4096 processes were used on 128 nodes, actual turnaround time due to queue waiting was considerably greater. A fixed time step of (1/64 + 1/128) = 0.234375 ms, i.e., an exact fraction of a power of 2, eliminated round off error in the integration of *dt* for long simulations and allowed spikes to be stored without round off error as binary single precision floating point values. The latter is important for using the spike train as the stimulus for retrospective simulation of a subset of cells on a desktop computer, in order to view any voltage or state trajectory. Visualization tools were created offline, with custom developed Python code using the Enthought Mayavi 4.3.0 environment (https://www.enthought.com/). Movies were created using spike time files to create individual frames, and a custom implementation of POVRay (www.povray.org) to generate in parallel high–quality 3D photo–realistic scenes. All frames were joined together into a compressed MPEG-4 movie using FFmpeg (www.ffmpeg.org). The production of a typical high-resolution frame (1920 ∗ 1024 pixels, about 0.7 Mb of data) required about 60 s of processing time.

**Table 2:** **Model parameters and execution times for a typical simulation**.

	**Seg (min-max)**	**States (min-max) (v, channels, and syn. gates)**		**Syn (min-max)**
MC (*n* = 635)	380,748 (189–1433)	5,259,735 (2536–20,028)		707,216 (308–2799)
GC (*n* = 69013)	4,344,724 (33–257)	26,892,317 (261–869)		707,216 (1–62)
Total	4,725,472	32,152,052		
	**Computation time**	**Comm. time (spike exchange)**	**Comm. time (multisplit)**	**Total run time (2048 procs)**
Average (sec)	27,149.35	68.53	555.94	32,552.86
Max (sec)	27,756.25	813.44	1453.96	

On a more technical note, which can be useful to readers interested in applying our methods to other brain systems, the otherwise excellent Allgather spike exchange method, which sends each spike to all processes, is not well suited for reciprocal synapse communication in the mitral-granule network because each spike initiated on one side of the synapse needs to be sent only to a single target containing the other side of the synapse. Furthermore as a spike propagates along a secondary dendrite, many reciprocal synapse threshold detectors are activated in a short time and thus there are several orders of magnitude more spikes generated per cell spike, thus requiring a substantial buffer transfer at minimum spike delay integration interval synchronization times. However reciprocal synapse communication is ideally suited to NEURON's Multisend method (Hines et al., 2011) which sends spikes only to the processes that have target synapses for them and does so while overlapping communication and computation. Explicitly, the average spike exchange time on 2048 BG/Q processes with 1.41 million synapses receiving a total of 745 million spikes in 40 s of simulation time was reduced to 69 s with Multisend (Table 2), from 5505 s with Allgather. Note that the distribution of cells on processes is chosen only to optimize load balance through use of the simple Least Processing Time (LPT) algorithm. LPT sorts all cell pieces according to decreasing computation time and each piece is placed successively on the process having the least total computation time. In particular, no effort is made to minimize MPI transmission paths between the two sides of a reciprocal synapse. Therefore, cell pieces can be considered to be essentially randomly located on processes. Also, it was not possible to obtain reasonable whole cell load balance with 4096 processes because the largest mitral cell has 20028 states (see Table 2) and, with a total model complexity of more than 31 million states, the balance is only 0.3 thus wasting 2/3 of the available compute time. However, by splitting off mitral cell secondary dendrite subtrees that connect to the soma (Hines et al., 2008), the largest piece has only 7139 states for an expected load balance of 0.95. A 0.98 load balance is typically obtained in simulations running on 2048 processors. Presently the network is constructed in parallel with a round-robin distribution of global identifiers, and the expected processing time needed by each cell piece is computed. Then, the proper load-balanced distribution is determined serially on rank 0, and the model is destroyed and re-constructed with the proper pieces on each rank. The total setup process currently takes 399 s on 2048 BG/Q processes and should be reducible to under 100 s by partial parallelization of the global identifier (gid) distribution phase and noting that a full build is not required to determine the number of states in each cell.

## Results

**Figure 1:**
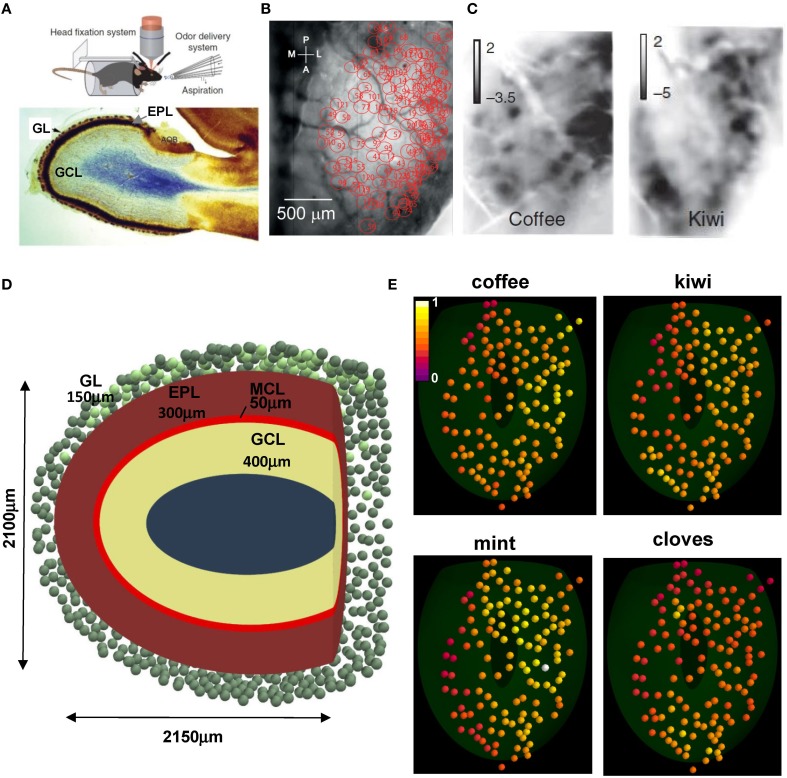
**Experimental constraints on glomerular location and activation. (A)** (top) The experimental setup to study glomerular activation in the dorsal surface of the olfactory bulb; (bottom) sagittal view of an olfactory bulb and its laminar organization; GL, glomerular layer; EPL, external plexiform layer; GCL, granule cell layer; **(B)** Location of the 127 activated glomeruli in the dorsal olfactory bulb included in this study; **(C)** Intrinsic optical signal imaging of evoked activity during presentation of two natural odors, coffee and kiwi; **(D)** Schematic organization of the olfactory bulb used for all simulations; GL, glomerular layer; MCL, mitral cell body layer; EPL, external plexiform layer; GCL, granule cell layer; the olfactory bulb volume was rendered in the {*x*, *y*, *z*} space as an ellipsoid defined by [(*x* − 50)/1050]^2^ + [(*y* − 12.75)/1200]^2^ + (*z*/1050)^2^ = 1, truncated along the main axis at 2150 μm; **(E)** Glomerular activation patterns, normalized for intensity, by several natural odors; the 3D location for the 127 glomeruli (colored spheres) was mapped from their 2D position in **(B)**; snapshots illustrate activation during presentation of the different odors (raw data kindly provided by A. Carleton). **(A)** (top), **(B,C)** are reprinted by permission from Macmillan Publishers: Vincis et al. (2012); **(A)** (bottom) is adapted from Elsaesser and Paysan (2007).

We start by illustrating the experimental setup and the patterns of glomerular activation in the dorsal surface of the olfactory bulb that are used in our model. Briefly, Figure 1A (top) illustrates the experimental setup, including the awake behaving mouse with head fixation, with optical intrinsic imaging of the dorsal olfactory bulb and the delivery of odor stimuli and clearing with aspiration (from Vincis et al., 2012). Below is a sagittal view showing the laminar organization of the olfactory bulb: the glomerular layer (GL, containing the glomeruli), the external plexiform layer (EPL, in which mitral and granule cell dendrites form their synaptic network) and the GCL (containing the GC bodies). From the Vincis et al. (2012) study of 30 odors we were provided with a random subset of 20 natural odor inputs to implement our model. These activated 127 unique glomeruli, spatially distributed in the dorsal area as indicated in Figure 1B (courtesy of A. Carleton). Typical intrinsic optical signal imaging of evoked activity during presentation of coffee and kiwi is shown in Figure 1C.

To relate our model of the glomerular patterns to the deeper mitral cell-granule cell network, we made a model of the laminar organization as shown in Figure 1D. This shows a tangential sagittal section of the olfactory bulb model (similar to Figure 1A, bottom), which we built as a truncated ellipsoidal shape of 1050 and 1200 μm semi-axes. This size is consistent with the adult mouse olfactory bulb (G Shepherd, unpublished observations). To estimate a reasonable distribution of glomeruli, a full set of 1800, 80 μm diameter, glomeruli (Royet et al., 1988) was initially randomly distributed in a 150 μm thick GL (green spheres); 127 of them, in the dorsal part (Figure 1D, light green spheres on the top) were mapped to those studied experimentally, based on their 2D position (Figure 1B). Their spatial location on the olfactory bulb surface implicitly determined the position of the 635 mitral cells soma (5 per glomerulus) used in our model, which were randomly distributed in the 50 μm thick mitral cell body layer (MCL) within an area approximately below (±100 μm diameter) the glomerulus to which they projected their tuft. Unless explicitly noted otherwise, in a typical network approximately 120,000 granule cell bodies were randomly distributed inside a 400 μm thick GCL. Each projected a single radial dendrite into the 300 μm thick EPL. Approximately 70,000 of them were connected to the mitral cell lateral dendrites, as discussed later.

Typical glomerular normalized activation patterns by several different natural odors are shown in Figure 1E. These illustrate several important features of the odor patterns. First, some patterns are quite extensive, as in mint; others are more restricted, as in cloves. Second, the extent of activation ranges from a high to low intensity, as in mint, to a limited range of intensity, as in kiwi. Finally, the specific sites and degrees of activated glomeruli are overlapping but different, consistent with virtually all studies and with the original finding and hypothesis that the different patterns can contribute to discrimination of different odors (Stewart et al., 1979).

This provides a model of the input patterns of glomerular activation that can be applied to any arbitrary pattern. In addition, it sets up the framework for relating any input pattern to the corresponding pattern of activation of the mitral cell-granule cell network. These data were directly used to drive the mitral cell-granule cell network activity.

**Figure 2:**
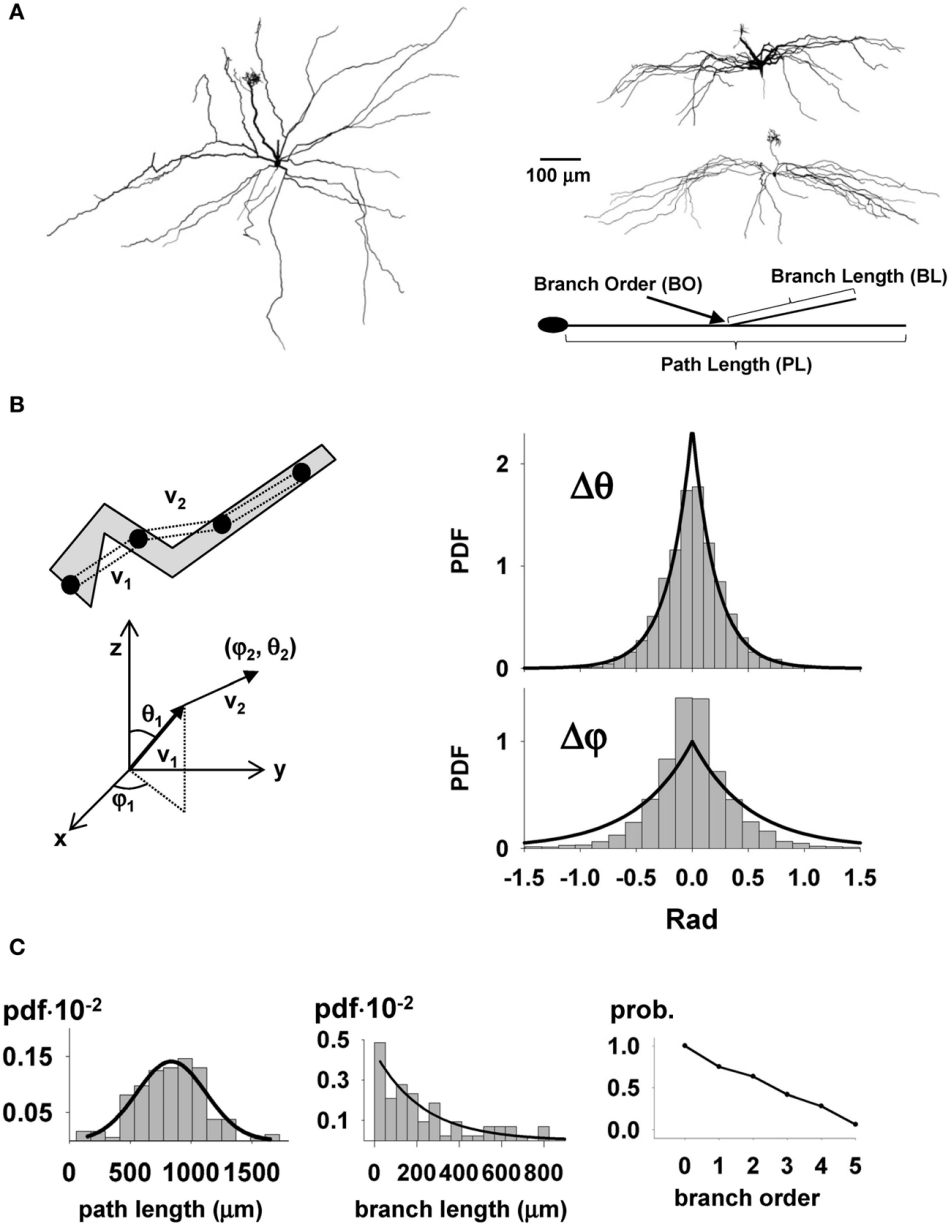
**Statistical parameters of full mitral cell 3D reconstructions. (A)** Typical reconstructed mitral cells (courtesy of K. Mori and K. Igarashi): left, overhead view showing full branching pattern of lateral dendrites and primary dendrite with terminal tuft; right, lateral views of two cells showing restriction of lateral dendrites to the external plexiform layer. The parameters used for the synthetic reconstructions are schematically illustrated in the diagram at bottom; **(B)** To calculate the average growing direction of a dendritic section, the raw data points composing the dendrites were first resampled in 20 μm sections, as schematically illustrated in the top left, and then the spatial orientation of each section was calculated in spherical coordinates (below); (right) Probability distribution function for the relative displacement in the spatial orientation of two consecutive membrane sections in spherical coordinates; The observed values for Δθ and Δφ were reproduced by the Laplace distributions PDF(Δθ) = 3.125 · exp(−|Δθ|/0.16) and PDF(Δφ) = 3.57 · exp(−|Δφ|/0.14), respectively; **(C)** Probability distribution function for dendritic path length (left), branch length (middle), and branch order (right); lines on each graph represent the functions used to reproduce the experimental distributions; for path length: PDF_PL_(*x*) = 0.0014 · exp(−1/2 · ((*x* − 838)/283)^2^), for branch length: PDF_BL_(*l*) = 0.0044 · exp(−*l*/0.0044).

The next step in our study was to set up the model of the mitral cells and their realistic cell-to-cell variability. The process is schematically illustrated in Supplementary Figure S1. For this scaled up model, we needed to provide for 635 different mitral cells, which is currently far beyond any available experimental data. We therefore used results obtained in a recent study by Igarashi et al. (2012) which provided us 8 stained and reconstructed mitral cells with full visualization of the entire dendritic trees (see Figure 2A). It should be stressed that this is the first set of such complete reconstructions that was available. The raw morphology files were first translated into NEURON format, and rotated along their principal axis. To calculate the spatial direction of a dendritic section, the raw data points composing the dendrites were resampled in 20 μm segments, as schematically illustrated in the top left of Figure 2B. An important feature that we considered was the growth direction of the dendrites (Figure 2B). The spatial orientation of each section was expressed in spherical coordinates (Figure 2B, bottom). The direction of a new dendritic segment was chosen according to the probability distribution function for the relative displacement in the spatial orientation of two consecutive membrane sections, Δθ and Δφ bar graphs in Figure 2B, right. For each new segment, the values for Δθ and Δφ were independently chosen using two Laplace distributions. Their parameters (see caption of Figure 2B), were found by a maximum likelihood method, and resulted in a statistically significant reproduction of the data (Mann-Whitney Rank Sum test, *p* = 0.061 and *p* = 0.144 for Δθ and Δφ, respectively). Since the main focus has been on the lateral dendrites, three additional main characteristics were analyzed in detail (schematically indicated in Figure 2A, right): the path lengths, the branch lengths, and the probability for each branch order (defined as the number of bifurcations from the cell body). The observed distributions of these features are shown in Figure 2C. The path and branch length distributions were generated using a Gaussian and an exponential function, respectively (see caption of Figure 2C), which gave a statistically significant reproduction of the experimental data (χ^2^ test, *p* = 0.269 and *p* = 0.292, respectively). The probability for each branch order was directly applied during the growth process.

**Figure 3:**
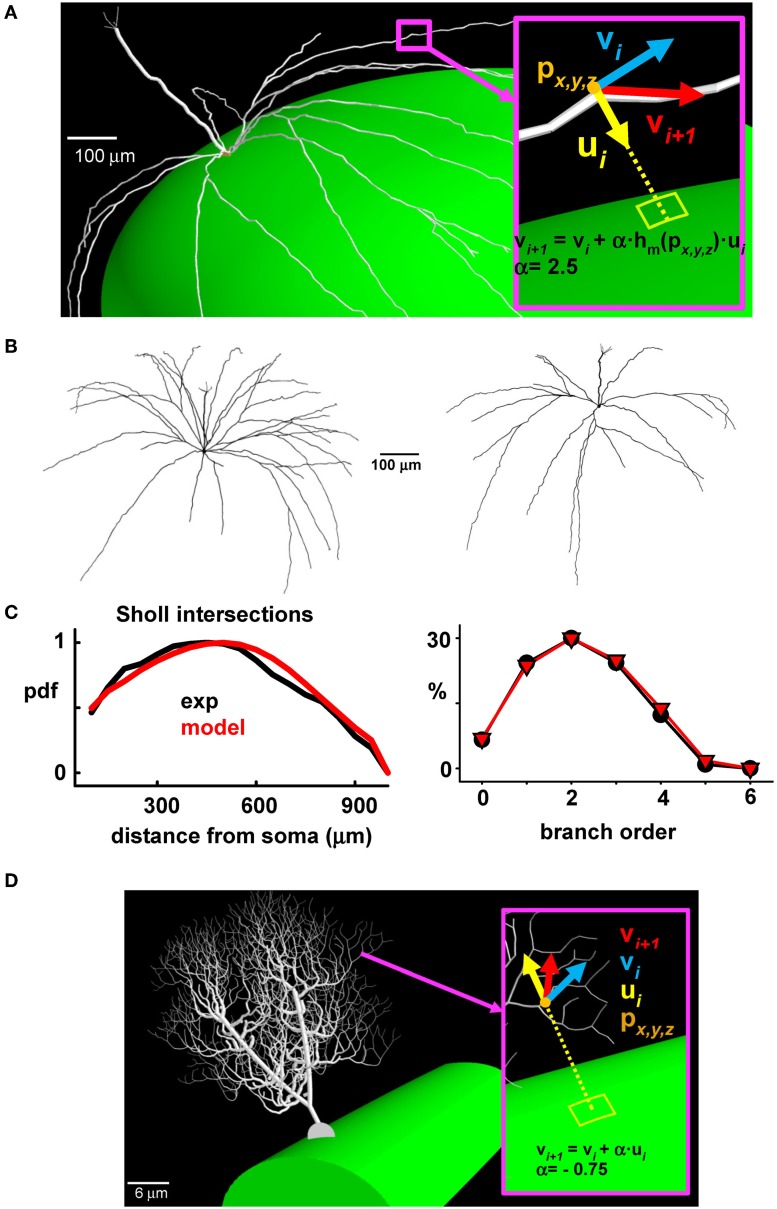
**The synthetic mitral cells are statistically indistinguishable from real reconstructions. (A)** To take into account the growing direction of the lateral dendrites, a correction factor was applied during the process of growing the lateral dendrites of a given mitral cell, according to their location (**p**_*x*, *y*, *z*_) on the olfactory bulb; the inset shows the vs. (**v**_*i*_, **v**_*i* + 1_) indicating the direction of the *i*-th dendritic section, the direction of the next section (*i* + 1), and the direction of the corrective factor, **u**_*i*_; the constant α and the function **h**_*m*_ (**p**_*x*, *y*, *z*_) depend on the system being modeled; **(B)** Typical synthetic 3D mitral cell reconstructions; **(C)** Sholl plot (left) and branch order probability distribution (right) from experimental reconstructions (black) and from modeled mitral cells (red); **(D)** The same procedure used to build synthetic mitral cells was applied to build a synthetic Purkinje cell, based on data from a 3D reconstruction (cell p19, downloaded from the neuromorpho.org public archive).

This provided a basis for implementing a general algorithm allowing the generation of synthetic morphologies for our full network of mitral cells (see Supplementary Figure S1). The generation of each new dendritic segment also needed to be consistent with the ellipsoidal surface of the olfactory bulb model. This was implemented by applying a correction factor to the lateral dendrites during their outgrowth according to their specific location in the olfactory bulb (Figure 3A). The final result is shown for two typical cells in Figure 3B. A Sholl plot (Sholl, 1953), typically used to compare the statistical properties of dendritic tree extension (Cuntz et al., 2010), was arranged to test the quality of the synthetic reconstructions. This type of plot (Figure 3C, left) reports the number of dendrites intersecting with the surface of a sphere centered at the soma, as a function of the sphere radius. An additional independent test was given by the proportion of dendrites of a given branch order (Figure 3C, right); both plots show the close correlations between experimental and model morphologies, which were statistically indistinguishable (Wilcoxon Signed Rank test *p* = 0.229 for the Sholl Plot and *p* = 0.313 for the branch order).

This approach thus gives a close approximation of modeled cells to experimental data, and it provided the basis to implement in a large population of mitral cells a cell-to-cell variability similar to that observed in the real system. The method is completely general; an example of its application to the case of a cerebellar Purkinje cell is shown in Figure 3D. In this case, the correction factor for growing the dendrites in the appropriate (molecular) layer was directed vertically, toward the surface (compare **u**_*i*_ in Figures 3A,D).

**Figure 4:**
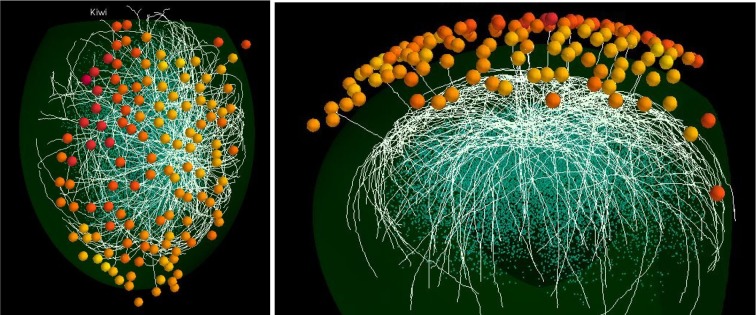
**Functional features of the mitral cell network.** Top (left) and lateral (right) view of a typical network formed by 18 mitral cells (white lines) projecting to different glomeruli (colored spheres); glomeruli are colored according to their activity during presentation of the kiwi odor; blue dots below mitral cell dendrites represent granule cell somas.

An example of the full model is shown in Figure 4. For clarity, only 18 mitral cells are shown. In the top view are seen the 127 olfactory glomeruli activated by the odor kiwi (cf. Figure 1D) and the underlying subnetwork of mitral cells. The white lines are mostly the lateral dendrites of the mitral cells spreading through the EPL. In the lateral view of this model, with the activated glomeruli above, several primary dendrites can be seen as white lines projecting to specific glomeruli. Beneath are the mitral cells with the lateral dendrites extending within the EPL as it curves around the ellipsoidal surface of the model olfactory bulb. Deeper are the cell bodies of the GC connected to the lateral dendrites of the subset of the 18 mitral cells shown here. Interactive 3D visualization tools to explore the network and results from a simulation will be available on the ModelDB website (acc.n. 151681).

The model thus provides the first integrated view of the full scale 3D architecture of a functioning olfactory bulb, and is focused on the experimental results obtained from the activation of the glomeruli in the dorsal surface. However, the method can easily be applied to any other arbitrary sites of glomerular activation, or to other types of inputs such as cortical feedback or centrifugal inputs from the basal forebrain or midbrain.

**Figure 5:**
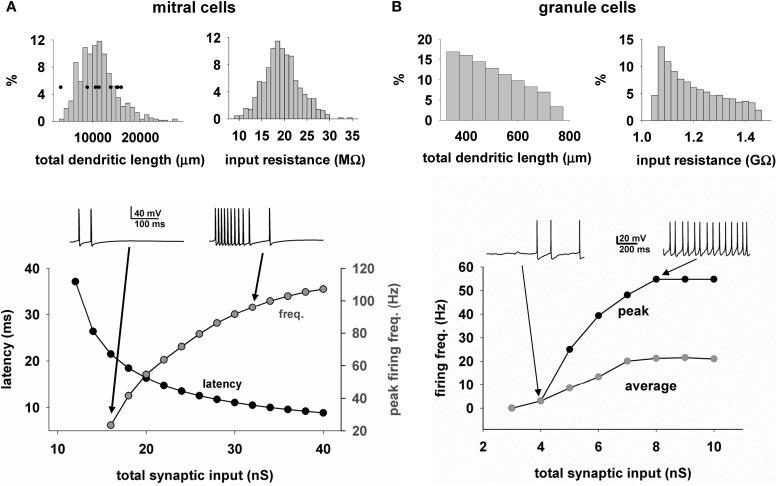
**Heterogeneity of mitral and granule cell properties. (A)** The distribution of the total dendritic length (top left) and input resistance (top right) for the mitral cells (*n* = 635); (bottom) First action potential latency (black symbols) and peak firing rate (gray symbols) of a typical mitral cell, as a function of odor input strength; traces above the plot illustrate the somatic activity during activation of the synaptic inputs modeling an odor input (single sniff); Black symbols in top left graph are the values obtained from the experimental reconstructions; **(B)** Distribution of the total dendritic length (top left) and input resistance (top right) for the granule cells (*n* = 69013); (bottom) Peak (black symbols) and average (gray symbols) firing rate of a typical granule cell, as a function of the input strength; traces above the plot illustrate the somatic activity during activation of 50 excitatory synapses randomly placed over the radial dendrite.

We next describe the properties of the mitral cells and GC that form the model network. One important characteristic of the modeled mitral cells was the total dendritic length of approximately 10,000 μm which determined the extent of interactions with GC. Another feature directly related to the cell excitability was the input resistance, which peaked at approximately 20 MΩ. These characteristics were in accord with experimental findings (Chen and Shepherd, 1997; Hovis et al., 2010). Two other important properties for odor processing are the latency of the first spike in response to odor input (Junek et al., 2010), and the firing rate (Shusterman et al., 2011; Smear et al., 2011). This is shown for the model of a typical cell in Figure 5A bottom, for a single simulated sniff as a function of the odor concentration, as measured by the total peak synaptic conductance activated on the mitral cell tuft. As in experiments, as the strength increases, the first spike latency decreases and the peak firing frequency increases. The ranges are in agreement with experimental findings (Cang and Isaacson, 2003; Egaña et al., 2005).

With regard to GC (Figure 5B, top), the total dendritic length was widely distributed. The granule cell bodies were distributed within the ellipsoidal GCL, which meant that their radial dendrites vary in length as they rise to the EPL, with a consequent variability in their input resistance. Below is shown the average and peak somatic firing frequency during random activation of 50 mitral cell synapses of increasing weights. In this graph, the peak frequency rises with increasing synaptic input as in the mitral cells, whereas the average frequency is shown reaching a lower level of firing rate, because the input due to the high level of synaptic firing, and corresponding high level of synaptic currents, saturates the granule cell responses in their dendrites.

**Figure 6:**
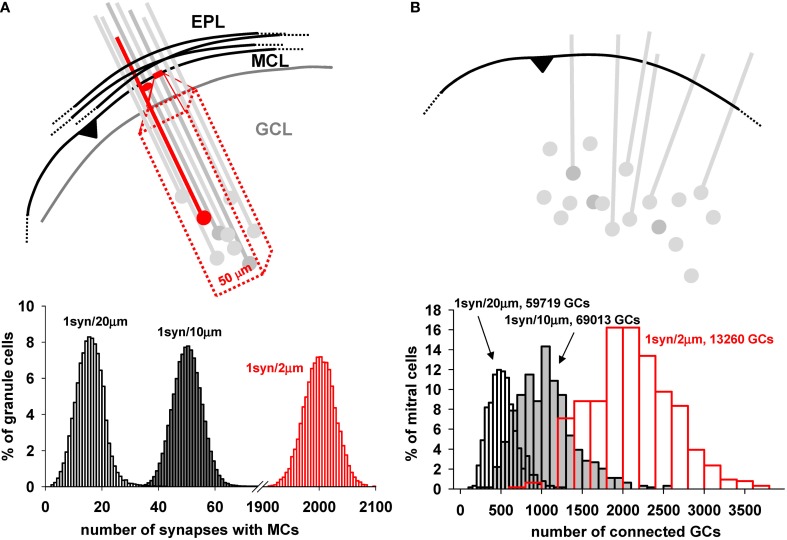
**Mitral cell—granule cell connectivity. (A)** Each dendrodendritic synapse was formed by selecting a dendritic mitral cell segment every 10 μm (on average) and randomly selecting a granule cell within a 50 μm rectangular volume deep to that site in the GCL, as shown in the diagram. **(B)** (top) Schematic representation of the connectivity from a mitral cell point of view; a given dendrite along its entire length connects to many more granule cells; Below each panel is reported the proportion of granule cells with a given number of synapses with mitral cells (left), and the proportion of mitral cells connected to a given number of granule cells (right) using 1 synapse per 2, 10, and 20 μm; Both plots were generated with a model including a total number of 122,166 (gray and white bars) or 16,013 (red bars) granule cells. Close to each graph is indicated the number of granule cells effectively connected for each case.

The functioning of the network depends critically on the connectivity between mitral and GC. The key factor providing for the connectivity is the dendrodendritic synapses between the mitral cell lateral dendrites and the spines of the GC in the EPL (Rall et al., 1966). The rule that nature uses to establish these synapses during development is unknown. To set up the initial arrangement of the network, we use an algorithm in which we fix the total number of GC in the GCL layer and the average number of synapses for a specific segment of mitral cell lateral dendritic membrane, one synapse for either 2, 10, or 20 μm of length. A schematic illustration of this process is shown in Figure 6A. For each segment of mitral cell dendrite, one granule cell is randomly chosen within a 50 μm rectangular volume inside the GCL (red box in Figure 6A). A spine is then added to the closest granule cell dendritic segment, and a dendrodendritic synapse is formed. The distribution of how many GC would be connected with a given number of mitral cells is shown in the plot in Figure 6A, bottom, for the assumptions of 1 synapse per 2, 10, and 20 μm of lateral dendritic length. It should be stressed this is the connectivity presumably set during development, corresponding to logical connections established between granule and mitral cells. The actual conductance of each synapse will be dynamically determined by its activity, according to the plasticity rule. Note that for the assumption of 1 synapse per 2 μm length, each granule cell forms on average 2000 synapses with mitral cells; with 1 synapse per 20 um length, the average is 16. This will allow testing simulations covering a wide range of possible connection density.

As a consequence of this connectivity rule, the number of mitral cells connected to a given number of GC is shown in Figure 6B. Above the diagram shows a single mitral cell with its lateral dendrites, potentially connected to a number of randomly placed GC and their dendrites. Below is plotted the proportion of mitral cells connected to a given number of GC, showing that the peak connectivity for a mitral cell could be easily adjusted in such a way to connect, on average, from ≈500 to ≈2000 GC, with a ratio ranging from about 20 to 100 GC per mitral cell. Thus, as can be seen from both plots in A and B, the actual overall density of connectivity can be adjusted in the model, to test presumably corresponding functional consequences. A change in synapse number and connectivity in the EPL during integration of adult-generated GC has been experimentally observed, and suggests that this mechanism can be an important variable for the network operations (Whitman and Greer, 2007).

**Figure 7:**
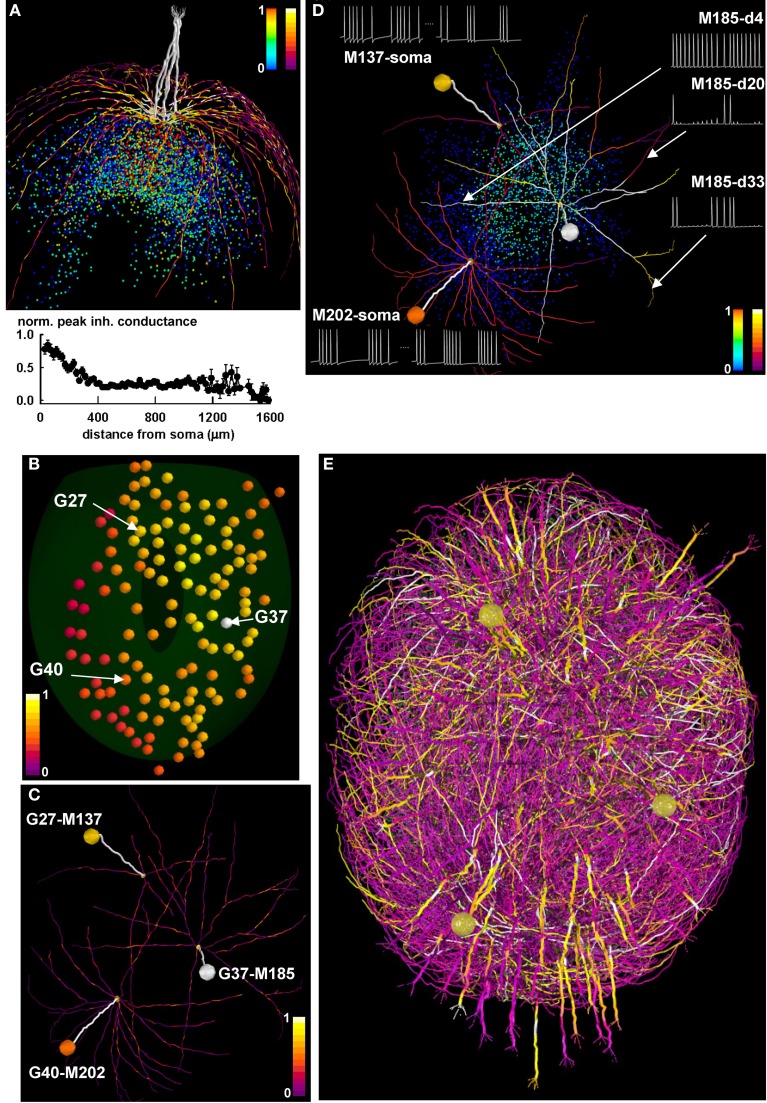
**Differential activity in mitral cell soma and dendrites depends on the spatial distribution of active granule cells. (A)** (top) the final configuration of the inhibitory peak conductance on mitral cell dendrites after a 40 s simulation of a network composed of only 1 glomerulus; dendritic segments are color-coded for the peak (normalized) inhibitory inputs they receive from granule cells, and colored points represent granule cell somas color-coded according to the peak inhibitory conductance of their synapses on the mitral cells; color scales on the top right of the panel represent normalized peak inhibitory conductance for granule cells (left scale) and for dendritic segments (right scale); note the formation of potentiated synaptic clusters, highlighted by bright colored dendritic segments and red granule cell soma; (bottom) normalized average peak inhibitory conductance on the lateral dendrites, as a function of the distance from soma; **(B)** Glomerular activation patterns for odor *mint*, color-coded for normalized intensity; the spatial locations of three specific glomeruli (G27, G37, and G40) are indicated by the arrows; **(C)** Three of the mitral cells (M137, M185, and M202) connected to the three glomeruli, with dendritic segments color-coded for the peak (normalized) inhibitory inputs they receive from granule cells, after learning *mint* odor; **(D)** The three mitral cells, with dendritic segments color-coded according to their average instantaneous firing activity at the end of the learning phase for *mint* odor. Colored points represent granule cell somas, and are color-coded according to the peak inhibitory conductance of their synapses on the mitral cells; traces on the right show typical 350 ms of membrane potential at different dendritic locations for M185; traces on the left show somatic potential of M137 and M202 during a 500 ms time window at the beginning and at the end of the learning phase for odor *mint*; the two time windows are separated by a dotted line; color scales on the bottom right of the panel represent normalized peak inhibitory conductance for granule cells (left scale) and for average instantaneous firing rate at dendritic segments (right scale); **(E)**. Snapshot of the response of the olfactory bulb system from a movie during presentation of the *mint* odor. A subset of the mitral cell dendritic network in the dorsal olfactory bulb is shown color-coded for membrane potential; inactive dendritic segments are shown in purple, action potentials in yellow. For the sake of clarity only one mitral cell per glomerulus is represented, except for glomeruli G27, G37, and G40, which are represented with all of their mitral cells. In all panels, color scales indicate normalized values. Glomeruli are colored according to the instantaneous spiking activity in the mitral cell tufts; red indicates no activity, yellow indicates spiking activity in any of the tuft dendrites.

In Figure 7 we discuss initial model findings and experimentally testable predictions. The first mechanism that was considered is the formation of a potentiated synaptic cluster. We have previously shown (Migliore et al., 2010; Yu et al., 2013) how clusters can be formed and directly compared with experimental findings (Willhite et al., 2006), using biophysically realistic but morphologically simplified mitral cells in a 1D space, which corresponds to that practically investigated with experiments in slices. However, the process of cluster formation cannot be taken for granted in the more complex morphological and topological network composed by mitral cells with realistic dendritic trees extending in the full 3D space. We thus carried out a simulation of a learning phase of a strong odor input, with 5 mitral cells projecting to a single glomerulus. The spatial distribution of the peak inhibitory synaptic conductance on the mitral cell dendrites (bright colored dendritic segments in Figure 7A, top), and the more quantitative plot for the average peak inhibitory conductance as a function of the distance from the soma (Figure 7A, bottom), demonstrate the presence of a population of GC with maximally potentiated synapses (cloud of red points in Figure 7A) close to the mitral cell somas. These results thus confirm that the formation of synaptic clusters is an extremely robust mechanism, which can be observed using a wide variety of biophysically realistic models (i.e., those able to reproduce the backpropagation of action potentials in the mitral cell lateral dendrites).

We then considered the glomerular activation during presentation of *mint* odor, focusing on the three glomeruli highlighted in Figure 7B, G27, G37, and G40. They are relatively far from each other and differentially activated by *mint*, with G37 and G40 receiving the stronger and the weaker input, respectively (compare their color coding). After the learning phase, the configuration of the peak inhibitory conductance on the lateral dendrites for three mitral cells projecting to these glomeruli (Figure 7C, M137, M185, M202) shows diffuse patches of relatively strong inhibition (shown as isolated bright colored segments), generated by the local interaction between backpropagating action potentials and GC. No potentiated synaptic clusters (i.e., groups of adjacent bright colored membrane segments, like those seen in Figure 7A) are observed in this case. The conditions under which synaptic clusters can be formed as a function of odor type, concentration, and temporal dynamics for natural odorants will be presented elsewhere (manuscript in preparation). The apparently random distribution of inhibitory inputs on the mitral cell lateral dendrites suggested exploring the firing activity in the different dendrites. This is an important issue, since the network self-organization (and thus the computational functions of the olfactory bulb) depends on dendritic, rather than somatic, activity. In Figure 7D we show the dendritic segments of the three mitral cells color coded for their firing frequency at the end of the learning period, and the peak inhibitory input elicited by each granule cell (Figure 7D, plotted as colored points on the background). For the most active mitral cell, M185, we found that different dendritic segments at approximately the same distance from soma can generate quite different action potential patterns (Figure 7D, see traces on the right). The other two mitral cells, M137 and M202, exhibit another surprising result. As can be inferred by comparing the darker color of M137 dendrites with that of M202 dendrites, after odor learning, M137 generates action potentials at a lower rate, with respect to M202. However, this is not what can be expected by looking at their respective glomerular activation, which is higher for M137 than for M202 (Figures 7B,D, compare colored spheres representing glomerular activation). Typical somatic traces of M137 and M202 at the beginning and at the end of the simulation (Figure 7D, traces on the left) illustrate the initial higher firing rate of M137, which becomes lower than M202 at the end of the simulation. As can be inferred by looking at the glomeruli activation in Figure 7B, this effect is caused by the higher mitral cell activity in the region of G27 with respect to that around G40 (the glomeruli to which M137 and M202 belong, respectively). This condition generates a stronger lateral inhibition on M137, revealed by the brighter colored GC.

The overall signal propagation over the entire bulb is illustrated in the movie snapshot in Figure 7E, where we represented the activity of all the mitral cells projecting to G27, G37, and G40, and one mitral for each of all other glomeruli. A full HD movie of the first 2 s of simulation can be downloaded from the ModelDB database, and a low-resolution version is shown as Movie 1. Taken together these results suggest that the underlying activity of the olfactory bulb circuits can be locally determined by the spatial distribution of both odor inputs and the long range of the mitral cell dendritic fields. Through their propagating action potentials the mitral cell lateral dendrites activate local granule cell inhibition of widely distributed mitral cell clusters. The 3D model thus reveals the critical role played by mitral cell lateral dendrites within the non-topographical organization of the olfactory bulb.

## Discussion

Implementation of an olfactory bulb model including full 3D realistic dendritic morphology for mitral cells represents a significant advance, adding a new level of possible investigations on the computational functions of this system: the possibility to implement the natural spatial location of odor inputs on the bulb; to investigate the long-standing problem of understanding the functional roles of the spatial arrangement of odor components; and a new approach to exploring the computational power gained by the independent propagation and modulation of activity on the multiple dendrites of the same mitral cell. These aspects are not accessible with previous models, and can now be studied in detail.

In addition to these insights into the olfactory bulb, this study identifies the general steps that can be used by computational modelers for building comprehensive 3D models of functioning microcircuits in the brain.

The first critical step is a representation of the input that is realistic, based on experimental data. To our knowledge, this is the first model of the mitral-granule cell network to include a realistic representation of the actual complex spatial pattern elicited in the GL by odor stimulation. This represents both the input from the olfactory sensory neurons and local processing by interactions in and between the glomeruli. As such, it is the initially processed odor representation that is driving the mitral-granule cell network. This knowledge of the input pattern is a unique advantage of the olfactory bulb compared with other brain regions. Our method is completely general, and can be integrated with more general approaches to other systems such as are being pursued in programs like the Human Brain Project or the Brain Initiative.

The second step is the method to synthesize a large population of cells. In our model, it involved the mitral cells with realistic morphologies growing on a curved surface such as the layers of the olfactory bulb. There are several methods published for growing neurons in 3D (e.g., Donohue and Ascoli, 2008; Zubler and Douglas, 2009; Cuntz et al., 2010; Wolf et al., 2013). Most of these are applied to generating single neurons or cortical columns. Our method is specifically aimed at the general problem of growing overlapping dendritic trees, and, in addition, within curved cortical layers. Although aimed at the olfactory bulb neurons, the method is completely general, as we show by growing cerebellar Purkinje cells (Figure 3). This should open the way to constructing realistic microcircuits with any arbitrary pattern of overlapping dendrites.

We show that the model of the pattern of glomerular activation can be combined with the model of the mitral-granule cell network in such a way to enable this relationship to be examined and analyzed in its natural architecture in full 3D. This view is not possible in the experimental situation. The model makes the experimentally testable prediction that the different dendrites of a given mitral cell can operate with firing patterns in ways that can be quite different from that at the soma. This is surprising because, in contrast with other large cortical pyramidal cell populations, mitral cell dendrites receive only a kind of “central excitatory input” from the soma.

The next step will be to test the hypothesis that there is a degree of specificity in mitral-granule interconnections. This would reflect a degree of organization of the olfactory glomeruli in relation to stimulating odor molecules, which in turn would reflect structural relations between the stimulating molecules and the binding pockets of the olfactory receptor molecules. At present this hypothesis is controversial. Some experimental evidence has been obtained for a regional grouping of glomeruli responding to structurally related odor molecules (Mori et al., 2006); other evidence has been obtained pointing to a near random distribution of responses to structurally related odor molecules (Soucy et al., 2009).

In addressing this problem, a recent theoretical study has compared lateral inhibition in a spatially continuous (retina-like) model with a distributed network model, and proposed that intraglomerular inhibition is the most likely mechanism to produce local inhibition independently of the distribution of the activated glomeruli and their associated mitral/tufted cells (Cleland and Linster, 2012). The authors are correct in emphasizing that locally generated inhibition is the key. Our model supports the evidence that local inhibition can also be generated at the last stage of the output pathway to the cortex by the mitral-granule cell interactions. Chen et al. (2002) showed that action potentials can propagate throughout the mitral cell lateral dendrites, reaching over 1 mm in distance, and can be modulated by local granule cell inhibition along the way. A series of computational studies (Migliore et al., 2007, 2010; Migliore and Shepherd, 2008; McTavish et al., 2012) has shown that the propagating impulse enables local inhibition to be activated onto mitral cells arbitrarily distant from the originating mitral cell. This implies that the mitral-granule cell network should be capable of producing the lateral inhibition of flanking members of a homologous series as originally suggested by Yokoi et al. (1995), independently of a need for close proximity of the activated glomeruli. A study of that operation using this model is in progress.

In conclusion, this 3D modeling approach provides a new basis for assessing the contribution of the mitral-granule cell network to processing of the distributed input from the olfactory glomeruli. It also provides a crucial advantage in addressing a critical aspect of microcircuit organization: the rules of connectivity between an output neuron and its interneurons. Connectivity may be conceived as formed during two steps; first during development, which is then continuously refined during subsequent activity-dependent processes. This is important not only in the olfactory bulb but also in other brain regions. The natural activity-dependent mechanisms depend on signal integration over the entire dendritic trees, which emphases again the need for 3D representation of the entire brain microcircuit.

### Conflict of interest statement

The authors declare that the research was conducted in the absence of any commercial or financial relationships that could be construed as a potential conflict of interest.
