# Supplementary material for: Distributed organization of a brain microcircuit analyzed by three-dimensional modeling: the olfactory bulb
Source: Front Comput Neurosci. 2014 Apr 29;8:50. doi: 10.3389/fncom.2014.00050 (PMC4010739; doi:10.3389/fncom.2014.00050)
Supplement: Supplementary file 2 [file DataSheet1.PDF]

Supplementary Fig.S1 for the paper "Distributed organization of a brain microcircuit analysed by three-dimensional modeling: the olfactory bulb" by M. Migliore et al. (2014).

Set Olfactory Bulb size, layers' boundary, and active glomeruli location;
Obtain NEURON files of full experimental **MC** 3D morphologies;
Rotate morphologies along principal axis;
Resample dendritic branches in 20μm segments;

Calculate range of **LD,** and distribution for **BL**, **PL**, **BO,** $\Delta\theta$**,** and $\Delta\varphi$ from data;
    Find parameters for distribution functions;

FOR each **MC**:
    Generate soma shape and location on **MCL;**
    Generate # of **LD** and direction of first segment of **LD** and **AD**;
    Create a list (**L**) of tip dendrites;
    ITERATE **N** times ***AND*** while **L** is not empty **DO**;
        FOR each element in **L**:
            test extension of a dendrite for **M** times:
                generate new segment (direction and diam);
                IF inside the boundary:
                    update L;
                    break;
            IF extension fails:
                delete element from L;
            ELSE **IF** can bifurcate:
                generate new tips and add to **L**;
            ELSE **IF** apical dend. is within **GCL**:
                generate first segment of **TD**s and add to **L**;

Abbreviations: MC, mitral cell**; LD**, lateral dendrites; BL, branch length; PL, path length; BO, branch order; MCL, mitral cell layer; AD, apical dendrite; GCL, granule cell layer; TD, tuft dendrites; N=1000; M=10.
